# Enhanced LED light driven photocatalytic degradation of Cefdinir using bismuth titanate nanoparticles

**DOI:** 10.1038/s41598-025-09184-8

**Published:** 2025-07-08

**Authors:** Sara Ishaq, Ahmed H. Nadim, Joliana F. Farid, Sawsan M. Amer, Heba T. Elbalkiny

**Affiliations:** 1 Faculty of Pharmacy, MSA University, October University for Modern Sciences and Arts, Cairo, Egypt; 2 Faculty of Pharmacy, Cairo University, Cairo, Egypt

**Keywords:** Photodegradation, Bismuth titanate nanoparticles, Cefdinir, Visible light, Wastewater samples, Environmental impact, Environmental chemistry, Pollution remediation, Sustainability

## Abstract

Photodegradation of antibiotics using visible light represents a promising approach for efficiently removing antibiotic contaminants from water sources. This study investigated bismuth titanate (Bi4Ti3O12) nanoparticles for the photodegradation of Cefdinir (CEF), a third-generation cephalosporin, under visible LED irradiation. Bismuth titanate nanoparticles were synthesized and characterized using transmission electron microscopy (TEM), X-ray diffraction (XRD), and diffuse reflectance spectroscopy (DRS). Factors affecting the degradation protocol were optimized using a central composite design model, and the degradation efficiency was assessed using a validated RP-HPLC method. Results of the experimental design demonstrated that bismuth titanate nanoparticles exhibited high photocatalytic performance (⁓ 98% photodegradation), which was found in an optimum condition of 0.05 g/L of BIT-NP in pH 5 for 50 µg/mL of CEF in 1 h at room temperature. The degradation efficiency depended on the concentration of the nanoparticles, the initial concentration of CEF, and pH. The antimicrobial effect of CEF was assessed before and after the degradation process, and the loss of antibiotic activity was observed after treatment. The findings provide valuable insights into developing innovative photocatalytic materials for the economic remediation of antibiotic-contaminated water sources using eco-friendly LED sources for degradation under visible light for the first time. This would offer a promising solution to mitigate the environmental impact of antibiotic residues.

**Supplementary Information:**

The online version contains supplementary material available at 10.1038/s41598-025-09184-8.

## Introduction

Access to safe and clean water sources is essential for a sustainable civilization. However, the rapid advancement of industrial processes has led to the release of various pollutants, including pharmaceutical chemicals, into water supplies^1,2] and [3^. These pollutants can have detrimental effects on both human health and the environment. Among these, antibiotics pose a significant concern due to their widespread use, which can increase aquatic toxicity, human toxicity, and resistance^4^. CEF (Fig. S1) is a broad-spectrum, advanced-generation cephalosporin antimicrobial agent widely prescribed for acute bacterial exacerbations of chronic bronchitis, community-acquired pneumonia, and other infections^5^. Due to its high consumption, traces of CEF can often be found in wastewater. Traditional wastewater treatment methods include pre-treatment, primary, secondary, and tertiary treatments, which aim to remove contaminants from water. However, novel nanomaterials are being explored to address the limitations of conventional methods. One promising technique for removing organic contaminants from wastewater is photocatalysis, an advanced oxidation process (AOP) that breaks down non-biodegradable pollutants with high chemical stability^6^. This study investigates the potential of photocatalysis using nanocomposites based on bismuth, specifically (Bi_4_Ti_3_O_12_) (BIT-NP), as an efficient and environmentally friendly NPs using visible light to reduce pharmaceuticals in water. Bi ions have been used in different forms as reported in^7^. Bi_4_Ti_3_O_12_ has unique physicochemical properties; such materials can absorb visible light due to their low bandgap, which is fundamental for solar-driven processes. The photocatalytic activity of these materials is further enhanced due to the increased mobility of holes and decreased rate of charge carrier recombination, which is achieved by the hybridization of the Bi 6s and O 2p orbitals in the upper valence bands. In addition, bismuth-based nanocomposites are chemically stable and less toxic, providing greener alternatives to conventional photocatalysts^8^. According to^8^, Bi ions may be stabilized effectively by constructing bismuth-based oxides and other semiconductor compounds; these not only reduce the environmental and health risks of free Bi ions but also exhibit enhanced photocatalytic and electronic properties, which are beneficial in applications such as water splitting and pollutant degradation. Through the immobilization of Bi ions into stable crystal lattices, their mobility and possible toxicity are minimized, thereby transforming a potentially toxic element into a useful and nontoxic component of advanced materials^9^. BIT-NP exhibits a large surface area, strong photocatalytic activity, enhanced electron mobility, and stability, making it a viable option for wastewater remediation. These nanoparticles are inexpensive and easy to synthesize. BIT-NP photocatalysts are activated by visible light (400 to 800 nm), offering a broader light absorption range than traditional TiO_2_, which is only active under UV light up to 400 nm^10^. This makes BIT-NP more efficient and eco-friendly. Previous studies have focused on using BIT-NP rather than pharmaceutical compounds for dye degradation. For example, A reported method employed titanium nanoparticles doped with silver to degrade dyes over a prolonged period^11^. Another method used was BIT-NP to degrade methylene blue^12^. The most often utilized NP before BIT-NP was titanium dioxide alone, or combinations such as TiO_2_ and ZnO, which have relatively wide bandgap energy as they absorb UV light and generate electron-hole pairs that can participate in photocatalytic reactions, but not as potent as BIT-NP^13^. Photodegradation under UV-A radiation of CEF, which is not eco-friendly, shows a disadvantage over the current study, which uses visible light and is more eco-friendly^14^. The current study focuses on the photocatalytic degradation of the cephalosporin antibiotic CEF using BIT-NP under visible light for the first time, showing a novelty using the central composite statistical design, a method combining factorial and response surface methodologies^15^. Central composite designs are advantageous for optimization because they can efficiently explore both the linear and nonlinear effects of factors, enabling accurate response surface modelling^16^. This study demonstrates the efficacy of BIT-NP in degrading CEF more effectively than previous methods, highlighting its potential for addressing antibiotic resistance and promoting cleaner water sources.

## Experimental

### Materials and chemicals

CEF (≥ 98%) was provided by Rameda Pharma, Egypt. Bismuth nitrate and titanium isopropoxide (≥ 99%) were supplied by Sigma Aldrich, USA. HNO_3_, NaOH, Ammonia, Anhydrous ethanol, and Methanol (≥ 99%) were provided by El Nasr Pharmaceutical Chemicals, Co., Egypt. Distilled water (DW) was used for the preparation and washing of samples. A 5 mg/mL stock solution was prepared by adding 0.05 g of CEF diluted to 10 mL of Methanolic NaOH (0.1 M) as a solvent in a volumetric flask. A working solution was prepared by transferring 2 mL of the previous stock solution and then diluting it to 100 mL using DW water to obtain a 20 µg/mL working solution.

### Instruments

A commercially available LED lamp 48 watts, 240 V, 4900 lumens was used in a closed chamber. Transmission electron microscopy (TEM) was performed at an accelerating voltage of 200 kV on a JEOL JEM-2100 high-resolution transmission electron microscope. X-ray diffraction (XRD) was analyzed using the XPERT-PRO Powder Diffractometer system (using Cu K α radiation). Diffuse Reflectance Spectroscopy (DRS) analysis was performed using UV-vis diffuse reflectance spectra (UV-vis DRS). Shimadzu UV-2550 spectrophotometer from 200 to 800 nm was used. HPLC Agilent 1200 series, with a photodiode array detector was used. The experimental design was performed using Design-Expert^®^11 (Stat-Ease Inc., Statistics Made Easy, Minneapolis, USA).

### Synthesis of BIT-NP

For the preparation of Bi_4_Ti_3_O_12_ crystals, the co-precipitation method was used^17^. Bismuth nitrate Bi (NO_3_)_3_·5H_2_O was dissolved in a 3 M HNO_3_ solution. As a modification, titanium isopropoxide C_12_H_28_O_4_Ti was added for the advantage of higher reactivity, solubility, and hydrolysis over the reported tetrabutyl titanate. In a 1:1 mixture solution of 3 M HNO_3_ solution and anhydrous ethanol (CH_3_CH_2_OH) titanium isopropoxide was dissolved under stirring at room temperature for 30 min. Bi (NO_3_)_3_ solution and C_12_H_28_O_4_Ti solution were mixed in a 12:1 mol ratio for Bi^3+^/Ti^4+^. Bi^3+^ concentration in the final solution was about 3 M. A dropwise addition of concentrated ammonia solution was added to adjust the pH to reach 10. White precipitate formation as a result was washed with dilute ammonia solution several times and then dried, smashed, and calcined at 500^◦^C. Consequently, a BIT photocatalyst was formed.

### Characterization analysis for BIT-NP

Transmission electron microscopy (TEM) was performed on JEOL JEM-2100 high-resolution microscope at an accelerating voltage of 200 kV, allowing the direct visualization of nanoparticles, providing information about their size and shape. The crystallography of NP patterns was detected by X-ray diffraction (XRD) using XPERT-PRO Powder Diffractometer system, with 2 theta (20° − 80°), with a minimum step size 2Theta: 0.001, and at wavelength (Kα) = 1.54614°. Diffuse Reflectance Spectroscopy (DRS) using JASCO Corp., V-570, Rev. 1.00 offers valuable insight into the optical properties and can assist in determining the bandgap of nanoparticles.

### Reversed-phase liquid chromatography

A previously reported method with minor modifications was applied^18^. Kromasil C_18_ (250 × 4.6 mm), 5 μm column was used. The optimum mobile phase was used which was composed of 0.1% tetramethyl ammonia hydroxide solution (pH5.5): methanol: acetonitrile: 0.1 M EDTA (5:3:2:0.4v/v) in isocratic elution at a flow rate of 1.0 mL/min. The wavelength was at 295 nm because of better degradation% results than the reported method wavelength. A standard solution of CEF (20 µg/mL) was prepared. Validation was performed per ICH Guidelines: Q2(R2)^19^. According to US Pharmacopoeia, system suitability parameters were calculated and interpreted from chromatogram peaks^20^.

### Photodegradation experiment

#### Screening of photocatalytic degradation

A 25 mL was withdrawn from the previously prepared working solution into 3 beakers adjusted to different pH (pH 5.0, 7.0, and 9.0). Before exposure to the LED lamp, the CEF and 0.025 g of BIT-NP mixtures were stirred in the dark for 30 min to achieve adsorption equilibrium left under a visible lamp in the closed chamber. After 1 h, samples were centrifuged and analyzed using HPLC assay (Fig. S2(a)) to check the NP photocatalytic degradation efficacy (Fig. S2(b)). In the presence of many factors such as pH, drug concentration, and the concentration of the NPs, optimization was obtained by preparing 3 working solutions in 1 h constant time factor. The following equation is used to calculate the degradation percentage of the antibiotic (D%):

$$\left( {{\text{D}}\% } \right){\text{ }}={\text{ }}\left( {{{\text{C}}_0}\, - \,{{\text{C}}_{\text{t}}}/{\text{ }}{{\text{C}}_0}} \right){\text{ }} \times {\text{ 1}}00.$$

As C_0_ is the initial concentration and C_t_ is the drug concentration after time t.

#### Data analysis (optimization of photocatalytic degradation)

The photocatalysis process analysis was performed using a quadratic central composite design, with the highest order polynomial and statistical analysis on the three responses (highest F-value and lowest P-value). Three factors were chosen for the polynomial model: NP concentration, drug concentration, and pH. The model was proposed as a function of observed response and independent variables. (Table 1) The three concentration levels of the NP were 0.01, 0.03 and 0.05 g/L. With levels of 50, 275, and 500 µg/mL of the drug concentration which is the second variable. pH values of 5.0, 7.0, and 9.0 were also selected. A total of 15 runs were planned and executed using operational variables.

**Table 1:** The factors and their levels used for CCD design.

Run	Factor 1 a: ph ca.	Factor 2 c: drug concentration µg/ml	Factor 3 b: NP concentration g/l	Response 1 degradation percent %
1	5	50	0.01	93.6
2	9	275	0.03	70
3	7	500	0.03	41
4	7	275	0.03	55
5	5	275	0.03	89
6	7	50	0.03	78
7	7	275	0.01	66
8	7	275	0.03	55
9	9	50	0.05	87
10	7	275	0.03	56
11	9	500	0.01	42
12	7	275	0.03	60
13	7	275	0.05	78
14	5	50	0.05	98
15	7	275	0.03	53.8

#### Batch experiment

A batch experiment to investigate the degradation of CEF at the optimized conditions using BIT-NP was carried out. Degradation of CEF (50 µg/mL) was performed in a cylindrical glass reactor with a working volume of 250 mL. A dose of 0.05 g/L of BIT-NP was dispersed in the solution, and the pH was adjusted to 5. The reaction mixture was continuously stirred at 300 rpm using a magnetic stirrer to maintain uniform suspension of the nanoparticles and ensure effective mass transfer. The reactor was kept at room temperature (25 ± 2 °C). Samples were withdrawn at regular intervals up to 1 h.

### Antimicrobial activity of BIT-NP

Using the qualitative disk diffusion method CEF activity following degradation protocol has been evaluated for antimicrobial effect by measuring the inhibition zone in mm against Escherichia coli ATCC 25,922, in Tryptic soy agar (BD Difco™) medium^21^. After the agar medium was sterilized, it was added to Petri plates and allowed to harden and solidify. A sterilized L-shaped glass loop was used to disperse fresh microorganism cultures across the media’s surface. A cylinder glass pipette of 5 mm diameter (sterilized) was used to bore cavities. Both the standard antibiotic (CEF only) and CEF sample after degradation with BIT-NP were placed serially in the cavities with the help of a micropipette and allowed to diffuse for 1 h. The standard antibiotic and solution solvents were used as controls. These plates were incubated at 37 °C for 18–24 h for antibacterial activity. The inhibition zone was measured and evaluated. Antimicrobial activity was measured in three replicates.

### Application to real surface water samples

Surface water from different points of the River Nile in the Zamalek area, Cairo was collected. Samples were then kept in a cooler till the transfer to the lab to avoid any contamination. Then, the suspended materials were removed by a 0.45 μm membrane filter. Samples were analyzed by the validated HPLC method to detect CEF levels (if any). Then, river water samples were spiked by 50 µg/mL CEF, and the optimized photocatalytic protocol was applied. Residual CEF concentrations were determined.

## Results and discussion

Implementation of visible LED light into photocatalytic degradation of antibiotics in wastewater has superior characteristics compared to traditional light sources. LED visible light uses less energy and has a longer lifespan, making it a more efficient and sustainable solution. Furthermore, LED light is readily adjustable to particular wavelengths, allowing for the targeted breakdown of antibiotics with the least amount of disturbance to other wastewater constituents. This innovative method not only enhances the degradation efficiency but also reduces the potential for the development of antibiotic-resistant bacteria, contributing to the overall protection of the environment and human health^22] and [23^.

### Characterization analysis of BIT-NP

#### Transmission electron microscopes (TEM)

**Fig. 1:**
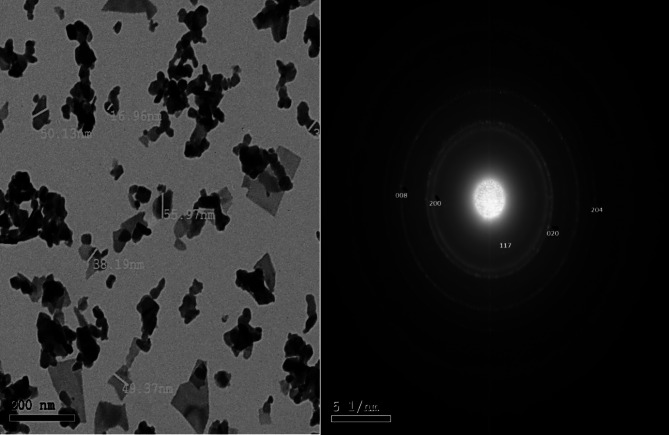
TEM micrograph of (BIT-NP). (**a**) TEM of BIT-NP showing a spherical-like shape NP of an average size of 40 nm. (**b**) Corresponding SAED (Selected Area Electron Diffraction) pattern displaying concentric rings, confirming the polycrystalline nature of the synthesized BIT-NP.

TEM images of BIT-NP revealed that the nanoparticles were mainly of spherical-like shape with relatively uniform distribution. The particles were found to be well distributed with very little agglomeration, indicating successful synthesis and stabilization of the nanostructure. As can be seen in (Fig. 1a), the average particle size of BIT-NP is approximately 40 nm, with most particles in a very narrow size range. This nanoscale order is characteristic of enhanced surface area, which is favorable for photocatalytic degradation. The corresponding SAED (Selected Area Electron Diffraction) pattern displays concentric rings, confirming the polycrystalline nature of the synthesized BIT-NP (Fig. 1b). The obvious contrast and sharp boundary revealed in the TEM micrographs also further confirm the crystallinity of the particles. The absence of large aggregates also suggests good colloidal stability, which is favorable for maintaining dispersion in aqueous suspension during photocatalytic reactions.

#### X-ray diffraction (XRD)

**Fig. 2:**
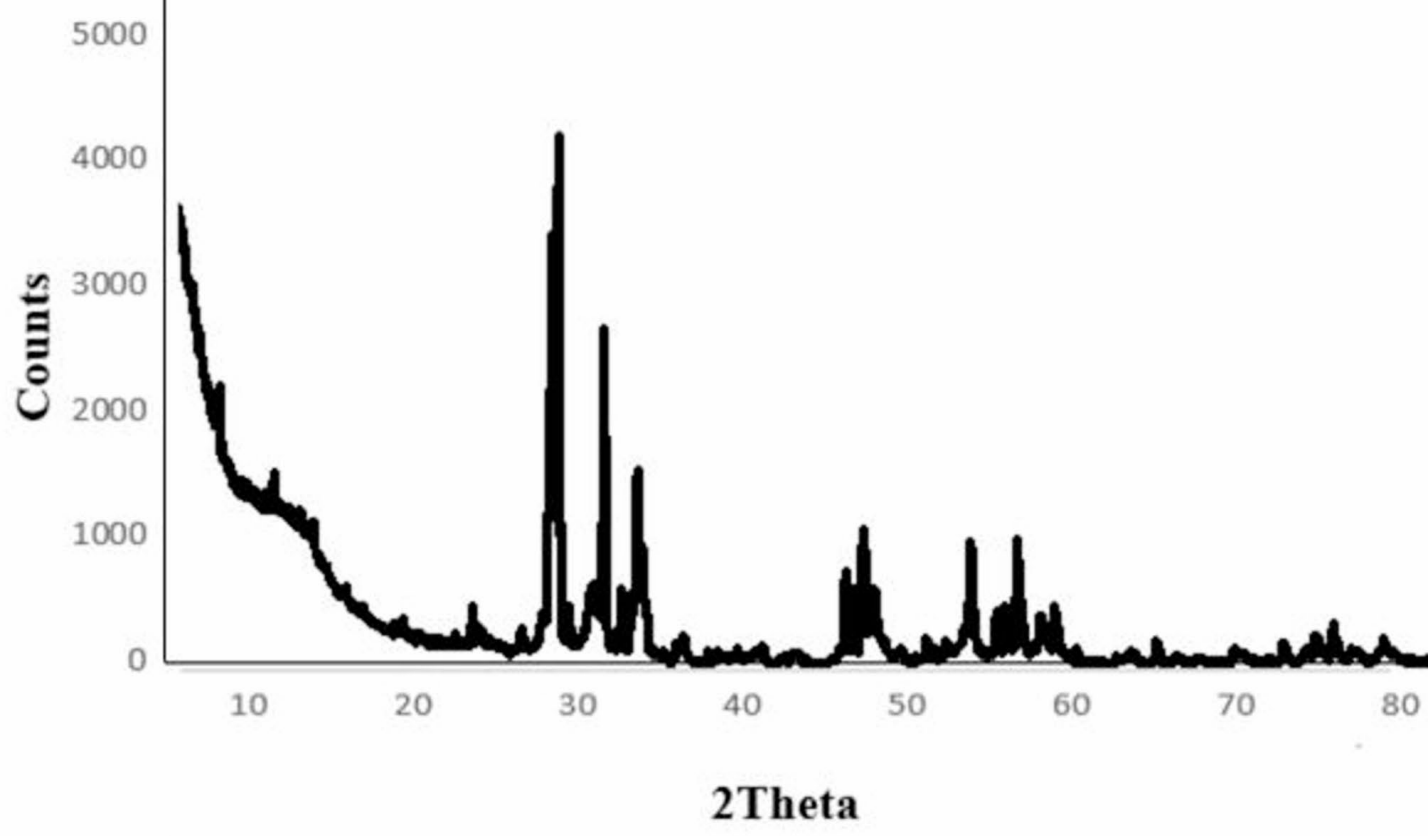
XRD of BIT-NP.

BIT-NP structure and its crystallinity were confirmed by exposing the samples to the XRD analysis, while the location of peaks will provide related information. Using the XPERT-PRO Powder Diffractometer system, it was performed at the wavelength (Kα) = 1.54614° (Fig. 2). with 2 theta (20° − 80°), and minimum step size 2Theta: 0.001. The observed sharp peaks indicated good crystallinity of the prepared samples. As depicted in the figure, the prepared samples included a single phase of layered perovskite Bi_4_Ti_3_O_12_. The XRD patterns were indexed based on the orthorhombic lattice (a = 5.45 Å, b = 5.41 Å and c = 32.82 Å)^24^.

#### Diffuse reflectance spectroscopy (DRS)

**Fig. 3:**
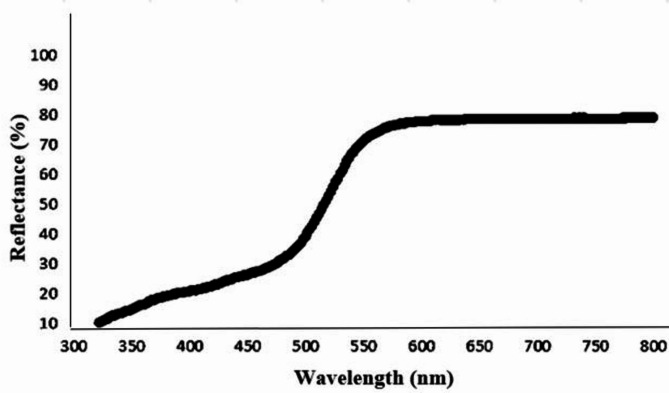
UV-Vis diffuse reflectance spectra of BIT-NP.

**Fig. 4:**
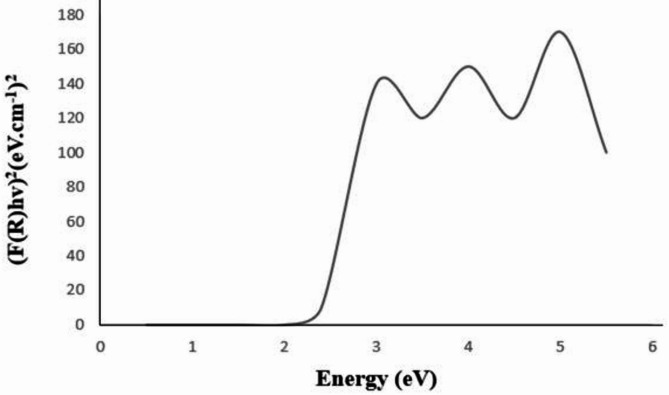
Band-gap energy of BIT-NP.

In the wavelength range of 400–800 nm, Fig. 3 shows the DRS spectra of BIT-NP. There was no absorption in the visible range, as indicated by the absorption edge of TiO_2_ was about 345 nm^25^. However, after the modification using BIT, its absorption edge is shifted to the visible range. Plotting the square of the Kubelka-Munk function against excitation Energy (eV) allowed for evaluating the optical band gap of the BIT-NP from diffuse reflectance spectra. As reported in^26^, the band gap energy of BIT-NP NPs was found to be 2.4 eV whereas the band gap energy of TiO_2_ was found to be 3.2 eV. Figure 4. shows that the band gap energy decreased after BIT modification, thus improving the photocatalytic activity of the photocatalysts under visible light.

### Reversed-phase chromatography

According to the reported chromatographic method^18^, the HPLC method was performed to determine the used samples’ concentrations.

A standard stock solution of CEF was prepared by dilution with the mobile phase and serial dilution was implemented to prepare different calibration solutions (2–500 µg/mL). Then, the validation parameters (linearity, precision, accuracy, specificity, ruggedness, robustness) and regression equation were summarized (Table 2). System suitability parameters were calculated according to US Pharmacopoeia^20^. ( Table 2).

**Table 2:** Summary of validation parameters and system suitability for the reported RP-HPLC assay.

Validation parameters:	
Accuracy (% recovery)^a^	99.10 ± 0.066
Linearity	2–500 µg/mL
Regression equation	y = 0.8715x + 0.7082
Regression coefficient (r)	0.9998
Precision (RSD%): Repeatability	0.57%
Sensitivity:	
LOD ^c^	0.64 µg/mL at 295 nm
LOQ ^d^	1.96 µg/mL at 295 nm
Robustness %RSD for different pH (± 0.2) ^e^	0.88%
Flow rate change (± 0.1 mL/min)	0.71%
System suitability parameters:	
Retention time (min)^f^	2.271
USP Tailing	1.15
Resolution (Rs)	2.4
Capacity factor (k’)	4.6
Selectivity	1.35

^a^Average percentage recovery of nine determinations over three concentration levels of (10-150-450) µg/mL.

^b^The intraday, average of nine determinations over three concentration levels repeated.

^c^LOD determined via calculation 3.3 (SD of the response/slope).

^d^LOQ determined via calculation 10 (SD of the response/slope).

^e^Average of nine determinations over three concentration levels.

^f^HPLC parameters reference values: K ≥ 1, α ≥ 1, Rs ≥ 1.5.

### Experimental design

Different variables influence the performance of photocatalysis. We studied various pH ranges, the first concentrations of all three medicinal products, and BIT-NP dose levels.

Fifteen experiments were designed by the central composite design model (CCD) using 3 factors and 3 levels. The range of variables for CEF and the effective parameters on the degradation of CEF BIT-NP are shown in Table S1. Then, experiments were accomplished, and the percentage of degradation was calculated. Results of the experimental design and the statistical analysis showed that CCD fitted the output to a quadratic model with the highest order polynomial. The model results obtained by experimental design are shown in the following equation:

$$\begin{gathered} {\text{Degradation percentage of CEF}}={\text{ }}+\,{\text{55}}.{\text{96}}--{\text{7}}.{\text{75 A }} - {\text{16}}.{\text{75 B}}\,+\,{\text{4}}.{\text{25 C }} - {\text{27}}.{\text{9}}0{\text{ AB}} \hfill \\ {\text{ }} - {\text{25}}.{\text{53 AC}}\,+\,{\text{27}}.{\text{7}}0{\text{ BC}}\,+\,{\text{23}}.{\text{54 }}{{\text{A}}^{\text{2}}}+{\text{ 3}}.{\text{54 }}{{\text{B}}^{\text{2}}}+{\text{ 16}}.0{\text{4 }}{{\text{C}}^{\text{2}}}. \hfill \\ \end{gathered}$$ Where (A) is the pH, (B) is the NP concentration, and (C) is the drug concentration.

**Fig. 5:**
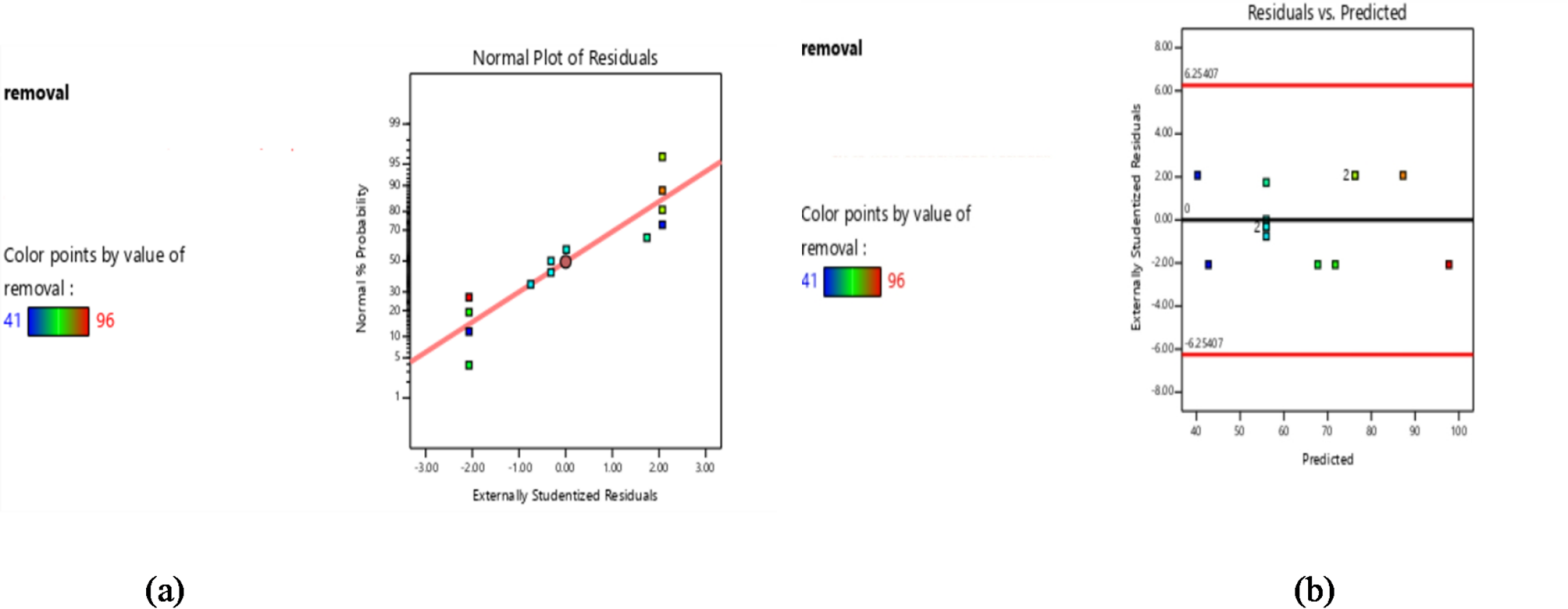
(**a**) Normal probability plot of residuals (**b**) Plot of the residuals versus the predicted response.

The model’s sufficiency was verified using the analysis of variance (ANOVA) as shown in Table 3. For CEF, quadratic and cubic models were chosen based on the experimental design. A high F-value of 53.46 and a very low P-value of 0.0002 for the model verify that the statistical model is significant enough to support minimal differences between the calculated and experimental responses. The study examined the destructive strength of CEF by photocatalyst, and all parameters such as drug concentration, BIT-NP concentration, and pH have significant P-values and are useful variables for this model. Since the difference is smaller than 2, the R^2^ value of 0.98 is quite near to the Adjusted R^2^ which is 0.97 indicating strong predictability. The adequate signal is shown by the adequate precision of 22.88. The model’s adequacy is evaluated to see if it provides a reasonable approximation to the actual system and if none of the assumptions of least squares regression are disobeyed. The normality assumption was verified by the normal probability plot of residuals, which produced a straight line as all of the points were on the diagonal. This suggested that the normality assumption was reasonable. (Fig. 5a). Furthermore, a random dispersion that appeared to be a constant variance was seen in the plot of the residuals against the predicted response (Fig. 5b).

**Table 3:** ANOVA model for optimization.

Source	Sum of squares	df	Mean square	F-value	*P*-value	
Model	4554.66	9	506.07	53.46	0.0002	significant
A-pH	160.17	1	160.17	16.92	0.0092	
B-NP conc	748.17	1	748.17	79.03	0.0003	
C-Drug conc	48.17	1	48.17	5.09	0.0737	
AB	257.95	1	257.95	27.25	0.0034	
AC	205.29	1	205.29	21.69	0.0055	
BC	254.27	1	254.27	26.86	0.0035	
A^2^	791.62	1	791.62	83.62	0.0003	
B^2^	17.90	1	17.90	1.89	0.2275	
C^2^	367.55	1	367.55	38.83	0.0016	
Residual	47.33	5	9.47			
Lack of Fit	24.50	1	24.50	4.29	0.1070	not significant
Pure Error	22.83	4	5.71			
Cor Total	4601.99	14				

### Design parameters affect and their interactions

**Fig. 6:**
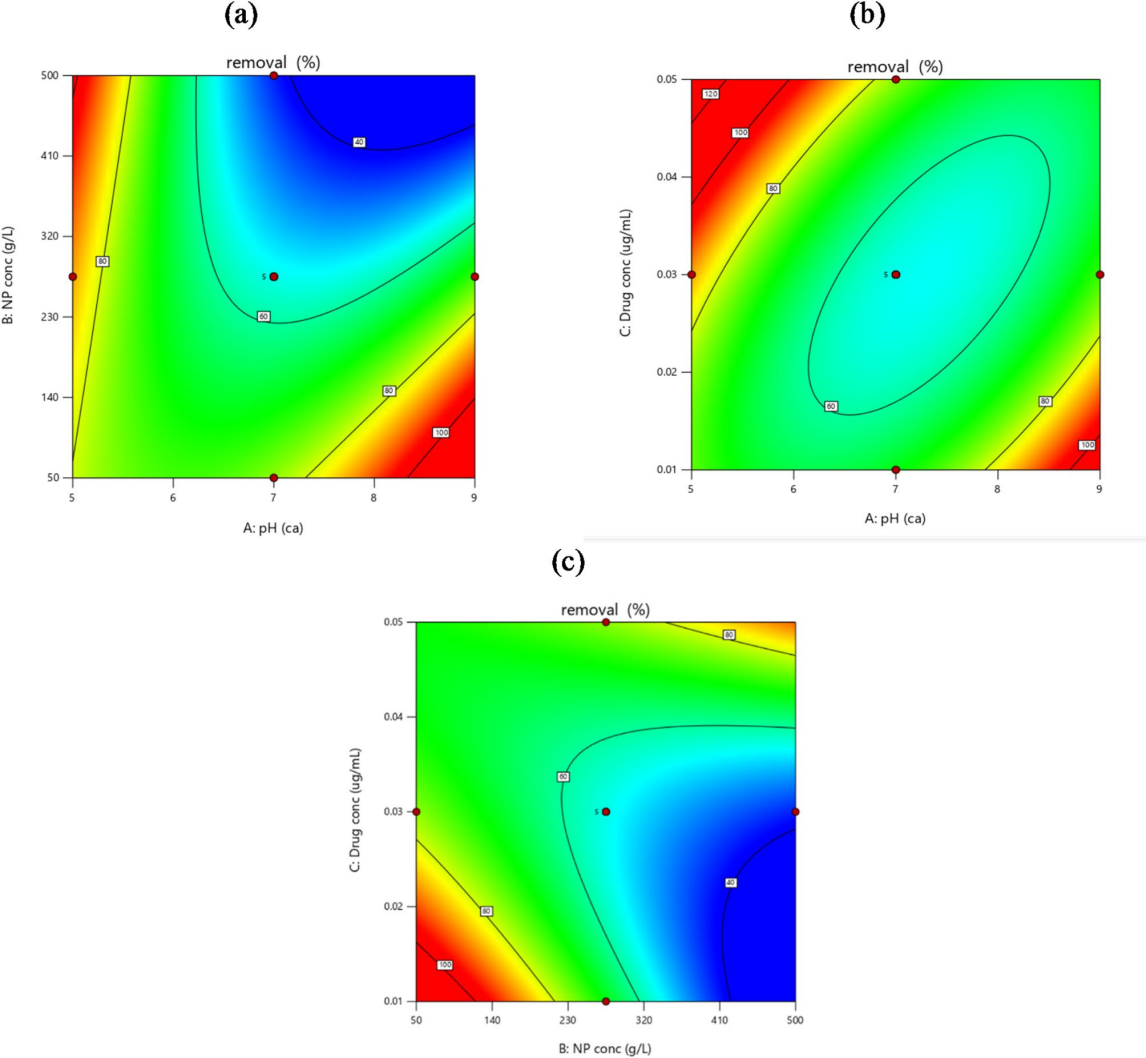
Contour plot of the removal percentage as a result of: (**a**) The effect of BIT-NP concentration versus pH. (**b**) The effect of CEF concentration versus pH. (**c**) The effect of CEF concentration versus BIT-NP concentration.

**Fig. 7:**
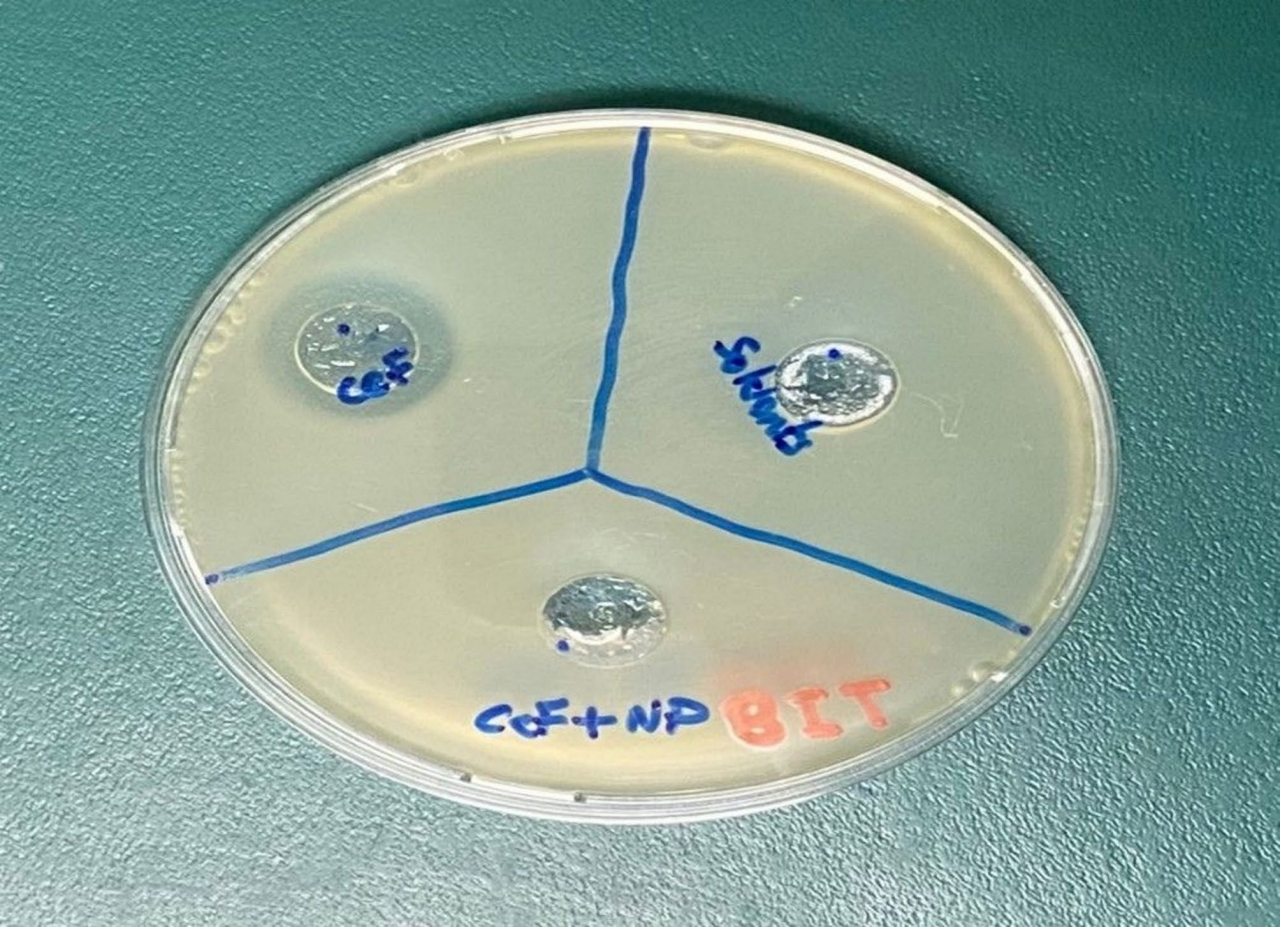
Inhibition of E. coli with standard CEF with no effect of used solvents and no effect with CEF after degradation with BIT-NP.

Contour plots illustrate the relationship between the three variables pH, photocatalyst BIT-NP concentration, and CEF concentration showing how each of the two components affects the other and how the photocatalysis process is affected (Fig. 6). As depicted in Fig. 7 The relationships between drug concentration and NP concentration (Fig. 6a), pH and NP concentration (Fig. 6b), and pH and drug concentration (Fig. 6c). A slight decrease in drug concentration showed a slight increase in the response of the degradation percentage, on the other hand, NP concentration (starting from 50 to 500 mg/mL) didn’t significantly affect the removal of CEF which shows a good advantage environmentally and low costs that it was a enough concentration to do the best degradation by light energy to drive pollutant degradation in photocatalytic degradation reaching up to 98% related to the previous conditions of the pH (factor a), BIT-NP concentration (factor b), and drug concentration (factor c). Choosing pH 5 for the optimum degradation of 98% is appropriate, as the pKa values of cefdinir (pKa 1.75 and 7.45) indicate that the group with a pKa of 1.75 will be ionized (negatively charged) at this pH. The second functional group, with a pKa of 7.45, will be neutral at pH 5. Therefore, at this pH, cefdinir will not exist predominantly as a neutral molecule; instead, it will be largely ionized due to the negatively charged group. At this pH, bismuth titanate nanoparticles are likely to exhibit a positive charge. This is because, in many metal oxides, including bismuth titanate, the surface tends to be protonated in acidic conditions, leading to a net positive charge^27^. The photocatalysis depends on the formation of free radicals such as [OH.] and [O_2_.] which are formed as a result of the absorption of the light into the catalyst when the catalyst absorbs the light it causes excitation to the electrons and forms a [O_2_.] these excitations leave a positive charge on the surface which form [OH.] due to the presence of water. Catalyst takes part in the reaction and speeds the transformation of organic compounds but remains in the unchanged form at the end of the catalyst cycle BIT-NP is a very powerful catalyst that is used with excellent electronic properties, high chemical stability, low cost, non-toxic and eco-friendly which is a positive point. This study shows many advantages over the reported methods due to the use of BIT-NP as a photocatalyst using LED visible lamp for the first time to break down CEF with a high degradation percentage of 98% in a short duration of 1 h only. The advantages of the study over reported studies^8,11,12,24,25] and [26^ are explained in Table 4.

**Table 4:** The advantages of the current study over the reported studies.

Removed matter	NP used	The drawback of the reported study over this current study	Citation
CEF	Candida Sp. SMNO_4_ and MgO Nanoparticles	Nano-bio integrated difficult synthesis system with an 88% removal percentage of CEF. The current study has more degradation%, easier preparation, and is more eco-friendly.	^29^
CEF	Immobilized TiO_2_ film	Photodegradation under UV-A radiation which is not eco-friendly shows a disadvantage over the current study	^14^
Tetracyclines	BIT-NP	A difficult, time-consuming hydrothermal synthesis of BIT-NP shows a low degradation efficacy of only 65% of tetracyclines.	^28^
Organic Compounds (dyes)	BIT-NP	The synthesized nanoparticles resulted in 77% degradation of the dye after 90 min UV irradiation, showing a low degradation %, long time, and UV non-eco-friendly irradiation.	^10^
Ceftriaxone (CEF alternative)	TiO_2_ and ZnO	degradation in much more time of 120 min and less deg% of 93%	^13^
Tetracycline Hydrochloride	BIT nanosheets with tunable crystal facets	Difficult synthesis of bismuth titanate nanosheets with tunable crystal facets with more heat, time, and effort compared to the current study	^30^

### Antimicrobial efficacy

As^21^ reported, the disk diffusion test proved that regular cefdinir (CEF) is of fine antibacterial activity against Escherichia coli ATCC 25,922 and has a distinct inhibition zone with a diameter of approximately 20 mm in an optimal concentration of 10 µg/mL. This is a testament to the effectiveness of CEF as a broad-spectrum antibiotic in normal conditions. In contrast, when Bi₄Ti₃O₁₂ nanoparticles (BIT-NP) were used for photocatalytic degradation of CEF, and the resultant solution was tested under the same conditions, there was no zone of inhibition observed. This absence of antibacterial activity confirms that BIT-NP had effectively degraded the active β-lactam structure of CEF to make it biologically inactive.

The experiment was carried out simultaneously with control antibiotics to verify the reproducibility of the antibacterial assessment. The loss of antimicrobial activity after photocatalytic treatment is irrefutable evidence of the capacity of BIT-NP to decontaminate pharmaceutical pollutants such as cefdinir. The finding is most pertinent in the context of remediation in the environment because it not just portrays the capacity of BIT-NP to break down leftover antibiotics in waste water, but also the capacity of BIT-NP to eradicate their biological effect, that is, a reduction of the risk for the formation of antibiotic resistance in aquatic environments. The results are summarized in )Fig. 7), showing the transition from an active pharmaceutical ingredient to a non-toxic byproduct upon treatment.

### Photocatalysis effect on real water samples

CEF was not detected in unspiked Nile River water samples, as its concentration was below the method’s limit of detection (LOD). To assess the photocatalytic performance of BIT-NP in a real water matrix, Nile River water was spiked with CEF at a concentration of 50 µg/mL and subjected to the optimized degradation conditions. The results showed a degradation efficiency of approximately 97.8%, which is comparable to that achieved in laboratory-grade distilled water under identical conditions. This indicates that the presence of naturally occurring organic matter and ions in the river water did not significantly hinder the photocatalytic activity of BIT-NP. Furthermore, when the developed degradation method was applied to actual environmental water samples containing CEF, the post-treatment analysis revealed negligible residual CEF, yielding results similar to those of the blank (uncontaminated) water samples. These findings confirm the robustness and effectiveness of the BIT-NP photocatalytic system for real-world applications in wastewater treatment.

### Conclusion

In conclusion, this study confirms the potential of Bi₄Ti₃O₁₂ nanoparticles (BIT-NP) as an efficient visible-light-driven photocatalyst for the degradation of cefdinir (CEF) in aqueous environments. Under optimized conditions (0.05 g/L BIT-NP, pH 5, 1 h), a high degradation efficiency of approximately 98% was achieved in both distilled and spiked Nile River water samples, indicating strong photocatalytic performance even in complex real-water matrices. The degradation process led to a significant reduction in antibacterial activity, as evidenced by the disappearance of the inhibition zone against E. coli, suggesting effective breakdown of the antibiotic structure. Morphological and structural characterizations confirmed that the synthesized BIT-NP were in the nanometer range and exhibited a polycrystalline nature, supporting their suitability for photocatalytic applications. Overall, the findings demonstrate that BIT-NP-based photocatalysis under visible light represents a promising approach for the removal of antibiotic contaminants from water sources, combining high efficiency, environmental compatibility, and applicability to real-world conditions. Despite the degradation efficiency, further work is necessary to evaluate the mineralization extent of CEF, identify intermediate degradation products, and test its potential ecotoxicity. The active species involved in photocatalytic degradation processes should be investigated. A long-term reusability and structural stability of BIT-NP in continuous flow systems should also be investigated to validate the practical feasibility of this method for real-world wastewater treatment.

## Electronic supplementary material

Below is the link to the electronic supplementary material.

Supplementary Material 1

## Data Availability

The authors confirm that the data supporting the findings of this study are available within the article and its Supplementary material. Raw data supporting this study’s findings are available from the corresponding author, upon request.
