# Supplementary material for: Enhanced LED light driven photocatalytic degradation of Cefdinir using bismuth titanate nanoparticles
Source: Sci Rep. 2025 Jul 8;15:24430. doi: 10.1038/s41598-025-09184-8 (PMC12238461; doi:10.1038/s41598-025-09184-8)
Supplement: Supplementary file 1 — Supplementary Material 1 [file 41598_2025_9184_MOESM1_ESM.docx]

**Supplementary materials**





Figure S1: Chemical structure of CEF.


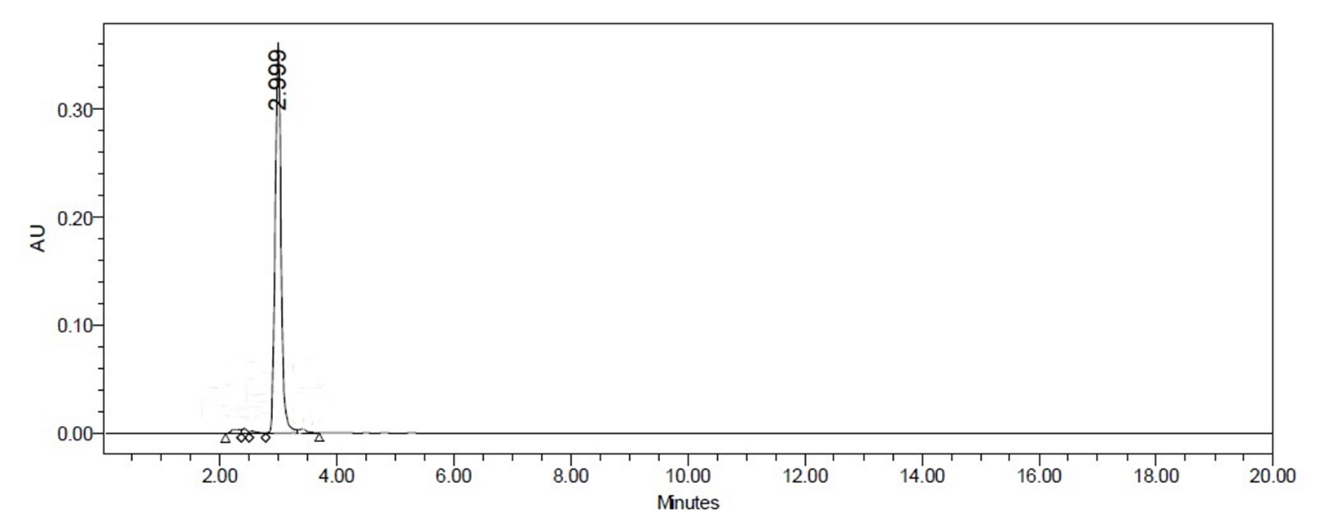


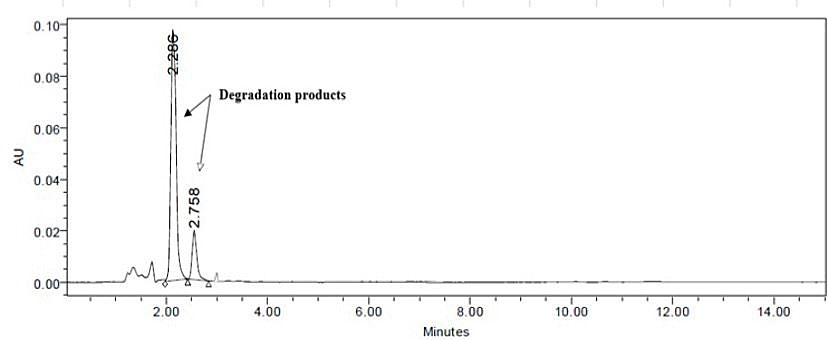


Figure S2. Chromatogram of standard CEF

(a) before degradation.

(b) after degradation.

Table S1. The range of input variables used in the response surface methodology for CEF.

| Factor | Name | Units | Minimum | Maximum | Coded Low | Coded High |
| --- | --- | --- | --- | --- | --- | --- |
| A | pH | ca | 5.00 | 9.00 | -1 ↔ 5.00 | +1 ↔ 9.00 |
| B | NP conc | g/L | 0.0100 | 0.0500 | -1 ↔ 0.01 | +1 ↔ 0.05 |
| C | Drug conc | ug/mL | 50.00 | 500.00 | -1 ↔ 50.00 | +1 ↔ 500.0 |
